# Carbenoxolone Disodium Treatment for Canine Pituitary-Dependent Hyperadrenocorticism

**DOI:** 10.1371/journal.pone.0166267

**Published:** 2016-11-08

**Authors:** Takahiro Teshima, Hirotaka Matsumoto, Tomoko Okusa, Rion Uchiyama, Hidekazu Koyama

**Affiliations:** Division of Therapeutic Science I, Department of Veterinary Clinical Medicine, School of Veterinary Medicine, Faculty of Veterinary Science, Nippon Veterinary and Life Science University, 1-7-1 Kyonan-cho, Musashino-shi, Tokyo, 180–8602, Japan; Colorado State University, UNITED STATES

## Abstract

Pituitary-dependent hyperadrenocorticism (PDH) is mainly caused by pituitary corticotroph tumors in dogs. A characteristic feature of corticotroph tumors is their resistance to negative feedback by glucocorticoids. In some animal species, including dogs, the aberrant expression of 11β-hydroxysteroid dehydrogenase (11HSD), a cortisol metabolic enzyme, is observed in corticotroph tumors. We previously reported that carbenoxolone (CBX), an inhibitor of 11HSD, suppressed ACTH secretion from the pituitary gland, and decreased cortisol concentrations in healthy dogs. Therefore, the aim of this study was to investigate the therapeutic effects of CBX on dogs with PDH. Six dogs with PDH were treated with 60 to 80 mg/kg/day of CBX for 6 weeks, followed by trilostane, which is a commonly used agent for canine PDH. CBX treatment led to a gradual decrease in both basal and in corticotropic releasing hormone (CRH)-stimulated plasma ACTH concentrations and CRH-stimulated serum cortisol concentrations, without side effects. However, basal and stimulated ACTH and cortisol concentrations remained higher than those of healthy dogs, and clinical symptoms such as polydipsia and polyuria were not ameliorated. After a 2-week wash-out interval, trilostane was administered for 2 weeks. Although basal plasma ACTH concentrations were higher after trilostane treatment than CBX treatment, polydipsia and polyuria resolved in all six dogs. The reason for the lack of improvement in polydipsia and polyuria with CBX treatment is unclear. Other mechanisms, in addition to a partial decrease in ACTH secretion, are likely to be involved. In conclusion, this is the first study to report the *in vivo* effects of CBX in dogs with PDH. The findings suggest that CBX inhibits ACTH secretion from canine pituitary tumors, resulting in lower cortisol concentrations.

## Introduction

Pituitary-dependent hyperadrenocorticism (PDH) is a common endocrine disease in dogs. In veterinary medicine, the most common treatment for PDH is medical management with trilostane or mitotane. The efficacy of these drugs has been well documented [1,2].

Glucocorticoid resistance, which is a characteristic of corticotroph tumors, is partially caused by abnormal expression of 11β-hydroxysteroid dehydrogenase (11HSD) in humans [3,4]. In human and murine corticotroph tumors, abnormal expression of both 11HSD type 1 (11HSD1), which converts inactive cortisone to active cortisol, and 11HSD type 2 (11HSD2), which converts active cortisol to inactive cortisone, has been reported [3–5]. We previously reported a similar pattern of abnormal expression in canine corticotroph tumors [6]. A previous study in murine corticotroph tumor cells found that carbenoxolone (CBX), an 11HSD inhibitor, improved the negative feedback effect of glucocorticoids under existing cortisol concentrations [5]. We also reported that CBX decreases plasma ACTH and serum cortisol concentrations via inhibition of 11HSD2 in healthy dogs [7], and that proopiomelanocortin mRNA expression and the ratio of ACTH-positive cells in the anterior pituitary were lower after CBX administration in healthy dogs. However, the *in vivo* effect of CBX on pituitary corticotroph tumors has not been reported in any species. Thus, we hypothesized that CBX could inhibit 11HSD2 in corticotroph adenomas, thus raising cortisol concentrations in these tissues. This increased cortisol level would in turn increase the negative feedback effect, decreasing ACTH secretion from corticotroph adenomas and thus lowering cortisol secretion from the adrenal glands. The purpose of this study was to determine whether CBX could suppress ACTH secretion enough to affect cortisol levels in dogs with PDH, as it does in healthy dogs.

## Materials and Methods

### Animals

All procedures involving client-owned dogs were approved by the Institutional Bioethics Committee of the Veterinary Medical Teaching Hospital at Nippon Veterinary and Life Science University (No. 26–4). Written informed consent was obtained from all owners.

Six client-owned dogs with PDH (3 spayed females, an intact male, and 2 castrated males) were included, consisting of 1 Pembroke Welsh Corgi, 1 Yorkshire Terrier, 2 Miniature Dachshunds, and 2 crossbreeds, aged from 7 to 12 years, with a body weight of 3.6 to 12.1 kg (Table 1). PDH was diagnosed based on clinical signs, routine laboratory examinations, endocrine examination (plasma ACTH concentrations and ACTH-stimulation test), abdominal ultrasonography, and magnetic resonance imaging of the pituitary. To obtain control values, six clinically healthy Beagles (age, 9.2±1.3 years; body weight, 9.9±0.8 kg) (ORIENTAL YEAST, Tokyo, Japan) were also included. The six control dogs were individually housed at the same laboratory animal unit in separate pens (1.1 x 0.9 m) in temperature-controlled rooms with a 12-h light: 12-h dark cycle. Dogs were provided with a small blanket and free access to water and were fed a commercial dry food twice daily (PROSTAGE Pork & Rice; Yeaster, Hyogo, Japan; 25.9% crude protein, 12.6% fat, 48.1% carbohydrates, 4.0% fiber). None of the dogs were had access to the outdoors during the experimental period, but they were allowed outside of their individual pens and could interact with the other dogs when animal care staff cleaned up their pens twice daily. All 12 dogs underwent a corticotrophin releasing hormone (CRH) stimulation test.

**Table 1:** Clinical characteristics of healthy Beagles and dogs with PDH.

Case^1^	Breed	Gender^2^	Age (yr)	Body weight (kg)	Pituitary height^3^ (mm)	P/B ratio^4^	ACTH^5^ (pmol/l)	pre-Cort^6^ (nmol/l)	post-Cort^7^ (nmol/l)	adrenal gland^8^	USG^9^
N1	Beagle	FS	10	9.4	4.7	0.28	3.5	116	337	normal	> 1.030
N2	Beagle	FS	11	10.0	4.3	0.26	3.3	105	326	normal	> 1.030
N3	Beagle	FS	10	9.5	4.3	0.27	2.9	80	344	normal	> 1.030
N4	Beagle	M	7	8.8	4.0	0.25	4.6	69	417	normal	> 1.030
N5	Beagle	MC	9	10.3	4.6	0.28	4.0	97	361	normal	> 1.030
N6	Beagle	M	8	11.2	4.4	0.25	5.3	113	342	normal	> 1.030
PDH1	Pembroke Welsh Corgi	FS	12	12.1	5.1	0.32	14.1	80	845	bilateral enlarged	1.010
PDH2	Crossbred	FS	11	7.6	6.1	0.38	24.0	342	1380	bilateral enlarged	1.008
PDH3	Yorkshire Terrier	MC	12	3.6	4.2	0.29	27.3	149	1007	bilateral enlarged	1.008
PDH4	Miniature Dachshund	M	7	8.7	8.3	0.67	50.1	406	1868	bilateral enlarged	1.006
PDH5	Crossbred	MC	8	6.5	6.2	0.53	40.9	174	886	bilateral enlarged	1.012
PDH6	Miniature Dachshund	FS	10	7.9	5.8	0.42	19.6	119	946	bilateral enlarged	1.008

^1^ cases: N1-N6: normal dogs; PDH1-PDH6: patients with pituitary-dependent hyperadrenocorticism.

^2^ sex: FS = female spayed, M = intact male, MC = male castrated.

^3^ pituitary height (mm) as measured on pre-operative enhanced T1-weighted transverse image.

^4^ pituitary height-to-brain area ratio × 10^−2^ mm^-1^ (P/B ratio ≤ 0.31 = normal sized pituitary, P/B ratio >0.31 = enlarged pituitary).

^5^ plasma ACTH (reference range 1.3 to 12.9 pmol/l); values at diagnosis of pituitary-dependent hyperadrenocorticism.

^6^ serum basal cortisol (reference range 17 to 132 nmol/l); values at 0min after intravenousadministration of 0.25mg of synthetic ACTH at diagnosis of pituitary-dependent hyperadrenocorticism.

^7^ serum post-ACTH cortisol (reference range 165 to 480 nmol/l); values at 60 min after intravenous administration of 0.25 mg of synthetic ACTH at diagnosis of pituitary-dependent hyperadrenocorticism.

^8^ pre-operative abdominal ultrasonography; enlarged = adrenal gland width > 7.5 mm.

^9^ urine specific gravity at diagnosis of pituitary-dependent hyperadrenocorticism.

### Study Protocol

After CRH-stimulation tests, all six dogs with PDH were treated twice daily for 6 weeks with encapsuled CBX (carbenoxolone disodium; LKT Laboratories, Inc., Saint Paul, MN, USA), administered orally with food. The dose of CBX was 60 mg/kg/day for the first 2 weeks, then 80 mg/kg/day for the following 4 weeks. Routine laboratory examinations, urine specific gravity measurement, and CRH-stimulation testing were performed on days 14 and 42. At 2 weeks after the end of CBX treatment (from day 56), all six dogs received trilostane (Adrestane, 3 mg/kg, SID; Kyoritsu Seiyaku Co., Tokyo, Japan) for PDH. After 2 weeks of trilostane treatment (on day 70), an ACTH-stimulation test was performed 4 hours after trilostane administration [8].

### CRH-Stimulation Test and Hormone Determination

Blood samples for hormone measurements were collected from the jugular vein and transferred to ice-chilled tubes containing EDTA and plain serum. Plasma and serum were separated by centrifugation at 4°C for 15 min and stored at -80°C until assayed.

The CRH-stimulation test was performed by collecting blood samples at 0 and 30 min after intravenous administration of ovine corticotropin-releasing factor (1.5 μg/kg; Peptide Institute, Inc., Osaka, Japan). All CRH-stimulation tests were performed 4 hours after administration of the morning dose of CBX.

Plasma ACTH concentrations were measured using a solid-phase, two-site chemiluminescent enzyme immunometric assay (Immulite 2000; Diagnostic Products Corporation, Los Angeles, USA) [9]. The intra-assay coefficients of variation (CV) were 9.6% and 4.9% at ACTH levels of 5.3 and 221.8 pmol/l, respectively. The inter-assay CV were 8.8% and 5.1% at ACTH levels of 5.8 and 248.9 pmol/l, respectively. Serum cortisol concentrations were measured using a competitive immunoassay (Immulite-Cortisol; Diagnostic Products Corporation) [10]. The intra-assay CV were 8.8% and 5.8% at cortisol levels of 74 and 524 nmol/l, respectively. The inter-assay CV were 10.0% and 6.3% at cortisol levels of 74 and 524 nmol/l, respectively. Serum cortisone concentrations were measured by a chemiluminescent immunoassay (DetectX Cortisone; Arbor Assays LLC, Ann Arbor, MI, USA) with duplicate samples [11]. The intra-assay CV were 5.7% and 7.9% at cortisone levels of 1.6 and 21.3 nmol/l, respectively. The inter-assay CV were 11.1% and 10.0% at cortisone levels of 1.8 and 21.3 nmol/l, respectively.

### Statistical Analysis

All results are presented as medians and ranges. Changes in ACTH, cortisol and cortisone concentrations, as well as cortisol-to-cortisone ratios and serum biochemical profiles were analyzed by two-way ANOVA, followed by Tukey-Kramer’s post hoc tests. Differences between control and after CBX treatment (at day 42) or trilostane treatment (at day 70) were determined by a two-sided Mann-Whitney’s *U* test. Statistical analyses were performed using Excel 2010 (Microsoft, Redmond, WA, USA) with the add-in software Statcel 3. Differences were considered significant for values of *P* < 0.05.

## Results

### Clinical Condition during CBX Treatment

**Fig 1:**
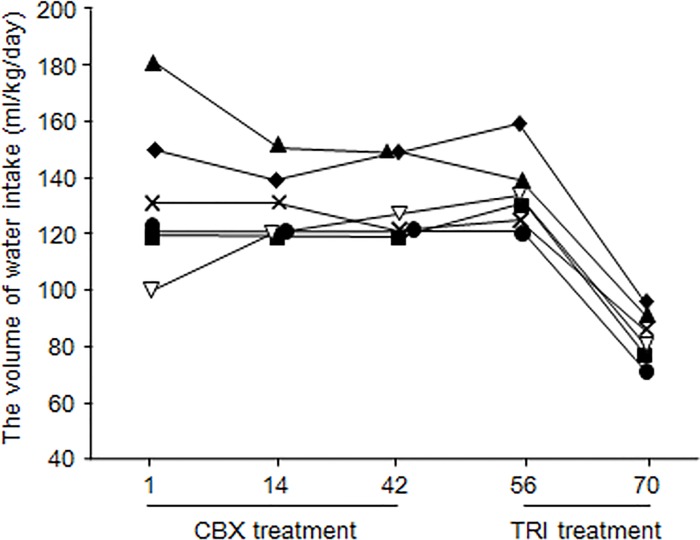
Changes in volume of water intake during CBX and trilostane treatment. Each line represents one dog with PDH: case 1 (■), case 2 (**◆**), case 3 (**▽**), case 4 (×), case 5 (▲), and case 6 (●). All six PDH cases were treated with carbenoxolone disodium (CBX) from days 1 to 42, and with trilostane (TRI) from days 56 to 70.

During CBX administration, adverse effects such as lethargy, anorexia, vomiting, and diarrhea were not observed in any dogs. Polyuria and polydipsia (PU/PD) were observed in all six dogs at diagnosis of PDH, and did not improve during CBX treatment (Fig 1). Serum biochemical profiles demonstrated that alanine aminotransferase (ALT), alkaline phosphatase (ALP), and total cholesterol (T-cho) levels decreased significantly during CBX treatment (*P*< 0.05) (S1 Table). Serum ALT and ALP levels did not decline to within the reference range, whereas serum T-cho fell to within the reference range during CBX treatment.

### Hormone Concentrations during CBX Treatment

**Fig 2:**
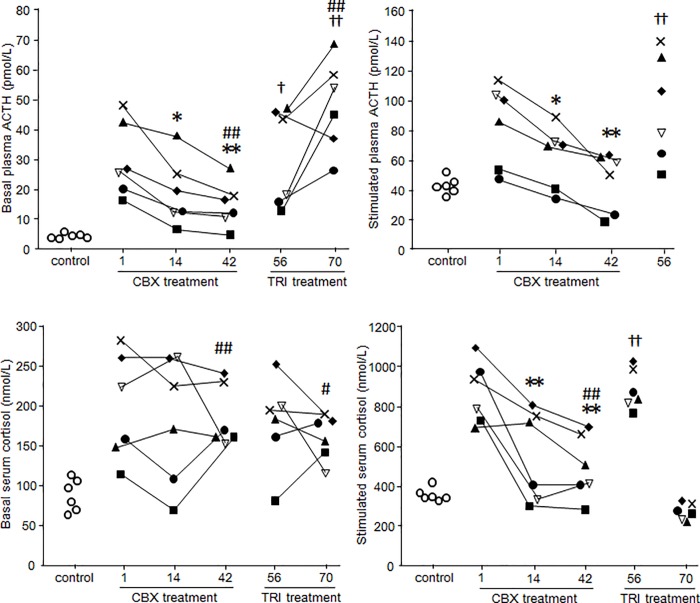
Changes in plasma ACTH and serum cortisol concentrations at basal and after stimulation during CBX and trilostane treatment. Each line represents one dog with PDH: case 1 (■), case 2 (**◆**), case 3 (**▽**), case 4 (×), case 5 (▲), and case 6 (●). White circles (◯) represent levels in healthy dogs. All six PDH cases were treated with CBX from days 1 to 42 and with trilostane (TRI) from days 56 to 70. Stimulated plasma ACTH and stimulated serum cortisol levels are after CRH stimulation on days 1, 14, 42, and 56, and after ACTH stimulation on day 70. *, *P*< 0.05 *vs*. day 1. **, *P*< 0.01 *vs*. day 1. †, *P*< 0.05 *vs*. day 42. ††, *P*< 0.01 *vs*. day 42. #, *P*< 0.05 *vs*. control. ##, *P*< 0.01 *vs*. control.

Basal plasma and CRH-stimulated ACTH concentrations decreased during CBX treatment (*P*< 0.05) (Fig 1), but remained higher than levels in healthy dogs. After initiating treatment with CBX, basal serum cortisol concentrations were unchanged, but CRH-stimulated serum cortisol concentrations decreased (*P*< 0.05) (Fig 1). However, CRH-stimulated serum cortisol concentrations also remained higher than levels in healthy dogs. Basal and CRH-stimulated serum cortisone concentrations fell with CBX treatment (*P*< 0.05) (Fig 2); the cortisol-to cortisone ratio was similar to that of healthy dogs.

**Fig 3:**
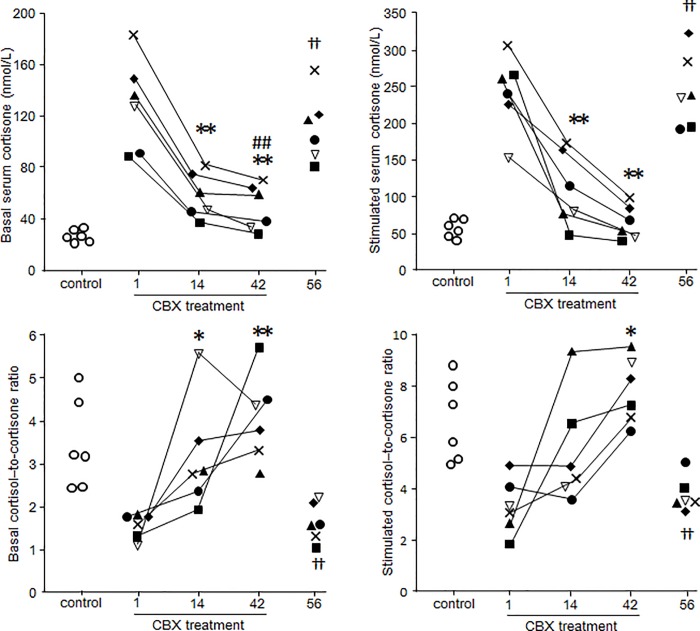
Changes in serum cortisone concentrations and cortisol-to-cortisone ratio at basal and after stimulation during CBX treatment. Each line represents one dog with PDH: case 1 (■), case 2 (**◆**), case 3 (**▽**), case 4 (×), case 5 (▲), and case 6 (●). White circles (◯) represent levels in healthy dogs. All six PDH cases were treated with CBX from days 1 to 42. Stimulated serum cortisone levels and cortisol-to-cortisone ratios are after CRH stimulation. *, *P*< 0.05 *vs*. day 1. **, *P*< 0.01 *vs*. day 1. †, *P*< 0.05 *vs*. day 42. ††, *P*< 0.01 *vs*. day 42. #, *P*< 0.05 *vs*. control. ##, *P*< 0.01 *vs*. control.

On day 56, before trilostane treatment, basal and CRH-stimulated ACTH and cortisol concentrations were higher than on day 42, and had returned to levels similar to those before CBX treatment (Fig 3).

### Clinical Condition during Trilostane Treatment

During trilostane treatment, PU/PD improved in all six dogs, less than 100 ml/kg/day of water consumed (Fig 1). ALT, ALP, and T-cho levels also decreased (*P*< 0.05), similar to the decreases seen with CBX treatment (S1 Table).

### Hormone Concentrations during Trilostane Treatment

At the onset of trilostane treatment (on day 56), basal plasma and CRH-stimulated ACTH concentrations were higher than at the end of CBX treatment. After trilostane treatment (on day 70), basal plasma ACTH concentrations were higher than at initiation of treatment (*P*< 0.05), but post-stimulation serum cortisol concentrations had fallen (Fig 2).

## Discussion

The principal cause of clinical signs in dogs with PDH is excessive cortisol due to ACTH secretion from corticotroph adenomas. In dogs, secretion of ACTH is episodic and pulsatile in nature, following a diurnal rhythm in healthy dogs and in those with PDH [12,13]. In addition, many types of stress can stimulate ACTH secretion. Therefore, we examined the secretory function in the pituitary by measuring plasma ACTH concentrations after CRH stimulation.

In this study, dogs with PDH were administered CBX at an initial dose of 60 mg/kg/day (30 mg/kg, twice daily), which was a dose selected to avoid side effects. This initial CBX dose has been shown to decrease plasma ACTH and serum cortisol concentrations in healthy dogs (S2 Table). After 2 weeks of CBX treatment at the initial dose, basal and CRH-stimulated plasma ACTH concentrations had decreased in all PDH dogs without any side effects, and CRH-stimulated serum cortisol concentrations had also decreased. However, PU/PD did not improve; therefore, the dose of CBX was increased to 80 mg/kg/day (40 mg/kg, twice daily) for 4 weeks. After increasing the dose of CBX, there was a further decrease in basal and CRH-stimulated plasma ACTH and in CRH-stimulated serum cortisol concentrations, but concentrations remained higher than in healthy dogs. Basal serum cortisol concentrations increased, but CRH-stimulated serum cortisol concentrations had fallen to levels similar to those of healthy dogs in three of six cases (PDH1, 3, and 6) at the end of CBX treatment. Nevertheless, PU/PD was not ameliorated in any of the dogs. In our previous study of healthy dogs, side effects were not observed with administration of higher doses of CBX [7]. However, because clinical signs in the present study did not improve with the increase in CBX dose from 60 to 80 mg/kg/day, we decided not to increase the dose further and instead switched to trilostane treatment. At initiation of trilostane treatment, ACTH and cortisol concentrations increased to levels similar to those before CBX treatment. After 2 weeks of trilostane treatment, ACTH-stimulated cortisol concentrations had decreased despite increased plasma ACTH concentrations, as previously reported [14], and PU/PD had disappeared in all six dogs.

In this study, CBX decreased ACTH secretion, but did not improve the clinical signs of dogs with PDH. There are several possible explanations for these findings. First, the effect of CBX on inhibiting ACTH secretion, and thus causing a subsequent decrease in cortisol concentrations, appeared to be insufficient. In all six dogs with PDH, both basal and CRH-stimulated ACTH concentrations decreased with treatment, but almost all cases continued to have higher levels than healthy dogs. Therefore, clinical signs such as PU/PD were not eliminated. In addition to the insufficient inhibition of ACTH secretion, other factors are likely to contribute to PU/PD, as indicated by the decrease in basal ACTH concentrations in case 1 to a level similar to that of healthy dogs. Second, because of its inhibitory effects on both 11HSD1 and 11HSD2 [15], there was a concern that CBX could suppress the conversion of cortisol to cortisone. The dose of CBX used in this study increased the cortisol-to-cortisone ratio. Thus, CBX inhibited 11HSD2 more than 11HSD1 in all six dogs with PDH. Cortisol is metabolized in the liver, with conversion to tetrahydrocortisol and then conjugation to glucuronic acid. Some cortisol is also converted to cortisone, which is metabolized in a similar manner, and then excreted in the urine [16]. Third, we speculated that prolongation of the elimination half-life of cortisol could reduce the rate of conversion of cortisol to cortisone. Therefore, the influence of cortisol may be greater than the apparent cortisol concentration. Fourth, CBX altered the balance of cortisol and cortisone in the kidney. During CBX treatment, the circulating cortisol-to-cortisone ratio improved to ratios similar to those of healthy dogs; however, the circulating ratio may not reflect renal levels. If conversion of cortisol to cortisone is suppressed, cortisol could interfere with binding of aldosterone to mineralocorticoid receptors and with the action of vasopressin [17,18], thereby inhibiting water resorption in the kidneys. In addition, CBX inhibits ACTH secretion from corticotroph adenomas by increasing negative feedback through suppression of 11HSD2; however, this suppression results in increased tissue cortisol levels in other organs as well. Therefore, clinical signs such as PU/PD did not improve despite lower circulating ACTH concentrations.

In conclusion, CBX inhibited ACTH secretion in dogs with PDH, without observable side effects; however, it did not improve clinical signs such as PU/PD. The reason for the lack of clinical efficacy was unclear, but it may be related to the insufficient impact of CBX in decreasing ACTH and cortisol concentrations. Further studies are required to determine the effects of CBX on cortisol metabolism and renal function in dogs with PDH, and to evaluate the effects of longer-term treatment and/or administration of higher CBX doses.

## Supporting Information

S1 Table Changes in serum chemistry during CBX treatment in dogs with PDH CBX, carbenoxolone disodium. TRI, trilostane. a, *P*< 0.05 *vs*. day 1. b, *P*< 0.05 *vs*. day 56.
(XLSX)

S2 Table Changes in basal and CRH-stimulated plasma ACTH and serum cortisol concentrations in healthy dogs. Five healthy dogs (5 intact males, aged from 1 to 3 years, with a body weight of 8.9 to 12.3 kg) were treated twice daily for 7 days with oral encapsulated CBX, administered with food. Data shown are % increase before versus after 7 days of CBX treatment. Data are presented as median (range). CBX, carbenoxolone disodium.
(XLSX)
